# Impact of Bovine Diet on Metabolomic Profile of Skim Milk and Whey Protein Ingredients

**DOI:** 10.3390/metabo9120305

**Published:** 2019-12-17

**Authors:** Jonathan B. Magan, Tom F. O’Callaghan, Jiamin Zheng, Lun Zhang, Rupasri Mandal, Deirdre Hennessy, Mark A. Fenelon, David S. Wishart, Alan L. Kelly, Noel A. McCarthy

**Affiliations:** 1 Food Chemistry & Technology Department, Teagasc Food Research Centre, Moorepark, Fermoy, P61 C996 Cork, Ireland; Jonathan.Magan@teagasc.ie (J.B.M.); Tom.ocallaghan@teagasc.ie (T.F.O.); Mark.Fenelon@teagasc.ie (M.A.F.); 2 School of Food and Nutritional Sciences, University College Cork, T12 YT20 Cork, Ireland; a.kelly@ucc.ie; 3 The Metabolomics Innovation Centre, School of Biological Sciences, University of Alberta, Edmonton, AB T6G1C9, Canada; jiamin3@ualberta.ca (J.Z.); lun2@ualberta.ca (L.Z.); rmandal@ualberta.ca (R.M.); dwishart@ualberta.ca (D.S.W.); 4 Teagasc Animal and Grassland Research & Innovation Centre, Moorepark, Fermoy, P61 C996 Cork, Ireland; Deirdre.Hennessy@teagasc.ie

**Keywords:** bovine diet, metabolome, amino acids, skim milk, sweet whey, acid whey, ideal whey

## Abstract

The influence of bovine diet on the metabolome of reconstituted skim milk powder (SMP) and protein ingredients produced from the milk of cows fed on pasture or concentrate-based diets was investigated. Cows were randomly assigned to diets consisting of perennial ryegrass only (GRS), perennial ryegrass/white clover sward (CLV), or indoor total mixed ration (TMR) for an entire lactation. Raw milk obtained from each group was processed at pilot scale, to produce SMP and sweet whey, and SMP was further processed at laboratory scale, to yield ideal whey and acid whey. The total amino acid composition and metabolome of each sample were analyzed, using high-performance cation exchange and a targeted combination of direct-injection mass spectrometry and reverse-phase liquid chromatography–tandem mass spectrometry (LC–MS/MS), respectively. The nitrogen composition of the products from each of the diets was similar, with one exception being the significantly higher nonprotein nitrogen content in TMR-derived skim milk powder than that from the GRS system. Total amino acid analysis showed significantly higher concentrations of glycine in GRS- and CLV-derived sweet whey and acid whey than in those from TMR. The cysteine contents of CLV-derived ideal whey and acid whey were significantly higher than for TMR, while the valine content of GRS-derived acid whey was significantly higher than TMR. The phenylalanine content of GRS-derived ideal whey was significantly higher than that from CLV. Metabolomic analysis showed significantly higher concentrations of the metabolites glutamine, valine, and phosphocreatine in each ingredient type derived from TMR than those from GRS or CLV, while the serine content of each GRS-derived ingredient type was significantly higher than that in TMR-derived ingredients. These results demonstrate that the type of bovine feeding system used can have a significant effect on the amino acid composition and metabolome of skim milk and whey powders and may aid in the selection of raw materials for product manufacture, while the clear separation between the samples gives further evidence for distinguishing milk products produced from different feeding systems based on LC–MS/MS.

## 1. Introduction

Skim milk powder (SMP) and whey typically form the basis of various dairy product formulations, particularly in infant milk formula (IMF) manufacture. Whey is obtained from the side-streams of other milk processing applications, such as cheese manufacture [1] or directly from skim milk by membrane microfiltration [2]. All essential amino acids (EAA) are present in whey [3], including branched-chain amino acids (BCAA), with high bioavailability relative to other dietary protein sources [4]. It is widely accepted that the concentration of amino acids (AA) in bovine milk is primarily influenced by genetic factors [5]. While previous studies have investigated the effect of bovine dietary supplementation of individual AAs on milk gross composition [6,7] and protein synthesis [8], little information exists on the potential effect of standard bovine feeding systems on the overall AA composition of milk. Recently, consumer interest has increasingly been focused on dairy products derived from what is perceived as a healthier [9], natural, or more sustainable, origin [10]. While previous research has demonstrated the effect of diet on the fat fraction of milk, the micronutrient composition of various protein ingredients is an important consideration in the manufacture of dairy products which aim to meet this demand. The relative abundance of low-molecular-weight metabolites may also provide an insight into verification methods for milk from different systems. The increasing prevalence of “grass-fed” product labeling will necessitate such verification methods.

The production of milk from cows fed outdoors on pasture is generally regarded by consumers as a more environmentally sustainable method, with distinct welfare advantages for cows free to forage naturally [11,12]. Despite this developing consumer interest, pasture-based production systems are estimated to represent only 10% of the global milk supply [13]. The dominance of this production system in Ireland and New Zealand is primarily attributable to their mild, temperate climates, with plentiful rainfall enabling long, consistent grass-growing seasons. Larger, more-intensive milk-producing industries in the Americas or Central Asia are almost exclusively based on indoor, concentrate-based feeding systems, allowing for more independence from climatic variances [12]. The extent of pasture grazing for milk production in the USA has been observed to decrease substantially with increasing herd size [14].

The application of quantitative nuclear magnetic resonance (1H-NMR)-based metabolomics to distinguish between rumen fluid and raw milk from pasture-based and total-mixed-ration-based feeding systems has previously been reported [15]. This nontargeted method has proven to be suitable for the purpose of verification of milk product origin, particularly in reference to claims of “pasture-fed” provenance. Reverse-phase liquid chromatography–tandem mass spectrometry (LC–MS/MS) is a highly sensitive analysis technique which utilizes liquid chromatography for the separation of compounds within a sample and subsequent compound analysis, using mass spectrometry. In targeted format, this method can be used for the detection and quantification of known compounds or metabolites, which can be identified from the established metabolome database for that sample type (e.g., milk metabolome). The potential to now extensively analyze the metabolome of dairy ingredients allows for greater understanding of the potential health effects from their consumption. Metabolomics has been demonstrated to offer potential as a mechanism for the verification of milk product origin claims [15].

The objectives of this study were to determine (1) the influence of perennial ryegrass (*Lolium perenne* L.), perennial ryegrass/white clover (*Trifolium repens* L.), and indoor total-mixed-ration-based feeding systems on the metabolome of SMP and whey protein ingredients and (2) the potential of LC–MS-based metabolomics for differentiating between milk and dairy product samples derived from different feeding systems.

## 2. Results and Discussion

### 2.1. Nitrogen Composition

Average concentrations of total nitrogen and nonprotein nitrogen (NPN) for SMP and whey powder samples are shown in Table 1. The total protein content of SMP and acid whey samples derived from both pasture-based systems were higher than those from the TMR system, while TMR-derived sweet whey and ideal whey samples contained higher total protein levels than samples derived from either pasture-based system. Nonprotein nitrogen content was highest in all TMR-derived ingredients and lowest in all samples derived from GRS. Indeed, the NPN content of TMR-derived SMP was significantly higher than that produced from the GRS-derived milk (Table 1). Increased NPN content has previously been observed in raw milk [16] and whole milk powder [17] derived from a CLV feeding system. Urea is the primary component of NPN [18] and products derived from the CLV system exhibit increased urea levels, resulting from the inclusion of nitrogen-fixing white clover in this system [15]. The increased concentrations of NPN observed in the TMR sample may arise from greater levels of dietary crude protein in the concentrate ration, which does not necessarily correlate with increased milk true protein content, but rather increased milk urea [19]. A study which analyzed the rumen fluid of cattle fed varying proportions of roughage in a total mixed ration diet found decreasing NPN content with increasing roughage content relative to concentrate content, though these figures did not differ significantly [20]. In the present study, increased average total protein content was observed in sweet whey samples derived from each feeding system, relative to native whey and acid whey, both of which were equivalent. The higher level of protein in sweet whey is likely attributable to the presence of soluble glycomacropeptide (GMP) resulting from the renneting process. Sweet whey derived from cheese manufacture contains 20–25% glycomacropeptide [21].

### 2.2. Total Amino Acid Composition

Average concentrations (g per kg total protein) for nineteen AAs in each ingredient type from each feeding system are shown in Table 2. The feeding system had a significant effect on concentrations of glycine, cysteine, valine, and phenylalanine in sweet whey, ideal whey, and acid whey samples. Concentrations of glycine in GRS-derived acid whey were significantly higher than those from TMR, while concentrations were significantly higher in sweet whey derived from both pasture-based systems when compared to TMR. Glycine is a nonessential proteinogenic AA, primarily utilized in the synthesis of collagen, with limited function in the synthesis of other proteins and metabolic pathways [22]. Previous work by Meléndez-Hevia and de Paz-Lugo [23] suggests that a restriction in the stoichiometry of the glycine synthesis reaction may lead to insufficient glycine production relative to metabolic demand, making glycine an essential or conditionally essential AA.

The CLV feeding system produced both ideal whey and acid whey with significantly higher concentrations of cysteine than the TMR system. Cysteine is a nonessential proteinogenic AA which, uniquely amongst the AAs, contains a thiol group. Cysteine also has a function in energy metabolism, along with some antioxidant capacity, owing to the affinity for redox reactions due to the presence of the thiol group [24]. Acid whey derived from GRS feeding had a significantly higher valine content than that from the TMR system. Valine, alongside leucine and isoleucine, is one of the three essential BCAAs [25]. It plays a structural role in the synthesis of globular proteins, where it forms the nonpolar center surrounded by polar residues [26], along with other metabolic functions, such as insulin secretion [27]. The only significant difference between both pasture-based feeding systems was that of the phenylalanine content of ideal whey, with GRS > CLV. Phenylalanine is an EAA which acts as a precursor for the synthesis of the nonessential AA tyrosine in the body. Both phenylalanine and tyrosine are utilized in the synthesis of amine-based hormones, such as dopamine, tyramine, and adrenaline, released within the body in stress responses [28].

The overall average values for each diet (combining each protein ingredient type) indicate a significant effect of feeding system over a wider range of AAs than in each ingredient type alone. These differences can be briefly summarized as follows: GRS > TMR for glutamine, alanine, and isoleucine; GRS and CLV > TMR for glycine and valine; CLV > GRS and TMR for cysteine; GRS > CLV and TMR for phenylalanine (*p* < 0.05). These effects are noteworthy, given the dominant influence of genetic factors in the total AA distribution of milk and the randomized distribution of cows into each feeding system in this study. Vanhatalo et al. [29] observed a significant effect of forage-feed type on plasma AA concentrations, with generally increased concentrations recorded for cows fed red clover silage in comparison to those fed grass.

Variations in the AA composition of each whey ingredient type are apparent when expressed as a combined average of the three feeding systems (Table 3). When compared to ideal whey and acid whey, sweet whey samples contained a significantly higher average concentration of the AAs most associated with the GMP component of cheese: isoleucine, proline, serine, valine, and threonine. Threonine, in particular, is an EAA present in high concentrations within GMP. Significantly higher concentrations of glycine, cysteine, and tyrosine were present in ideal whey, when compared to sweet whey and acid whey, while the histidine content of acid whey was significantly higher than both other whey types. While the total protein content of the SMP samples is markedly higher than that of each whey type, increased values for cysteic acid, taurine, aspartic acid, glycine, alanine, and cysteine were observed for each whey type when compared to SMP, owing to concentration of these AAs in the whey protein fraction of milk protein. Gorissen and Witard [30] reported similarly increased relative concentrations of glycine, cysteine, alanine, and aspartic acid in whey protein, when compared to skimmed milk and particularly casein. The protein used in the manufacture of IMF is selected on the basis of the amino acid profile that best mimics that of human milk. Variations due to feeding in the amino acid profile of skim milk powder and whey-protein powders, which typically form the primary ingredients for conventional IMF production, would be a noteworthy consideration for IMF manufacturers.

### 2.3. Metabolomic Profiles of Protein Ingredients

LC–MS analysis identified 46 individual metabolite compounds (47 in total) in SMP and whey protein samples, 25 of which were free AAs, including 19 of the 20 standard proteinogenic AAs. The average concentration of each metabolite is shown for SMP, sweet whey, ideal whey, and acid whey in Supplementary data Tables S1–S4, respectively, and as an overall average by feeding system in Table S5. Glutamic acid was the most abundant free AA in all samples from each feeding system. Glutamic acid has previously been shown to be the free AA present in the highest concentration in milk [31,32].

Four compounds were found to be significantly different between diets across the four ingredient types and are shown in abbreviated form in Table 4. In each of the four ingredient types, concentrations of glutamine in the TMR sample were significantly higher than both the GRS and CLV samples. Glutamine is generally regarded as a nonessential AA, although it has recently been suggested to be considered conditionally essential, following investigation of stress-response requirements [33]. It is primarily utilized in the biosynthesis of proteins, with additional functions in glycogen synthesis and the maintenance of the intestinal mucous membrane [33]. Concentrations of serine in GRS-derived SMP, sweet whey, and ideal whey samples were significantly higher than in those derived from TMR. Serine is regarded as a conditionally essential AA, which, like most other AAs, plays a role in protein synthesis, with additional functions in cell proliferation, hepatic gluconeogenesis [34], and immune response [35]. Similarly, significantly higher concentrations of phosphocreatine were observed in the TMR sample in each of these three ingredient types. Phosphorylation of the endogenous AA creatine occurs within muscle tissue by the action of creatine kinase [36]. Phosphocreatine is integral to adenosine triphosphate generation within the muscle and subsequent control of muscle contraction [37]. Concentrations of valine were significantly higher in TMR-derived SMP, ideal whey, and acid whey than both pasture-based systems. This is notable, given the lower concentrations of valine in TMR samples in the aforementioned total AA analysis, which may imply a greater proportion of total valine in the GRS and CLV samples is present in the bound form. While the concentrations of most total AAs were lower in the TMR-derived samples than both the GRS and CLV samples (Table 2), the inverse can be seen for the concentrations of most free AAs in the metabolome analysis (Supplementary data Tables S1–S5). As free AAs are constituents of the nonprotein nitrogen component of milk [38], the overall increased concentrations of free AAs in the TMR samples in each product may be correlated to their increased nonprotein nitrogen contents (Table 1), which represent a greater proportion of the total nitrogen of these samples than those from GRS or CLV. This trend may also suggest that, while the quaternary structure of milk proteins assembled from amino acids transferred to the bovine mammary gland may be genetically determined, the concentrations of free amino acids in the serum phase of milk (i.e., aqueous phase containing whey proteins) may be influenced by dietary interventions. In comparison to the other protein ingredients, acid whey samples exhibited greater variation in the average concentrations of a number of metabolites. Average concentrations of acetylornithine, alpha-aminoadipic acid, and leucine in acid whey were 3 to 4 times higher than the other ingredient types, while concentrations of tyrosine were extremely low or absent in comparison to the other products.

Metabolomic analysis of raw milk produced from cows on the three feeding systems used in this study, using 1H-NMR (O’Callaghan et al.) [15], identified 11 metabolites in common with this study: aspartic acid, betaine, choline, creatine, creatinine, isoleucine, glutamic acid, leucine, proline, tyrosine, and valine. Among these compounds, a significant effect of feeding system was found for concentrations of betaine, choline, creatinine, proline, tyrosine, and valine (Supplementary Data, Table S5) in both studies. Of all metabolites identified in the present study, choline was present in the highest concentrations in all samples, though average concentrations for GRS samples were significantly higher than those from TMR (Supplementary Data, Table S5). A similar result was observed for raw milk by O’Callaghan et al. [15], although the inverse was found in rumen fluid samples with significantly higher choline concentrations observed in TMR samples than in both GRS and CLV samples. Choline is considered an essential nutrient both in bovine [39] and human [40] nutrition, wherein it has a number of important functions, such as phospholipid biosynthesis and neurotransmitter synthesis via conversion to acetylcholine [40]. In cattle, dietary choline is susceptible to extensive ruminal degradation and must be supplemented in a rumen-protected form [41], while the majority of choline secreted into milk is the product of de novo synthesis of phosphatidylcholine [42]. It is therefore unlikely that the differences in choline content observed between milk and whey produced from cows from each feeding system can be attributed to direct absorption and transfer of choline from each feed type. Rather, it may infer that the differences observed are attributable to modulation due to diet of the rumen microflora (in this case, protozoa), as was previously suggested by O’Callaghan et al. [15], leading to variation in rates of de novo choline synthesis. José, Santos & Lima [42] suggest that a high-concentrate diet may reduce rumen protozoa numbers, leading to a decrease in the availability of choline to the cow.

**Figure 1:**
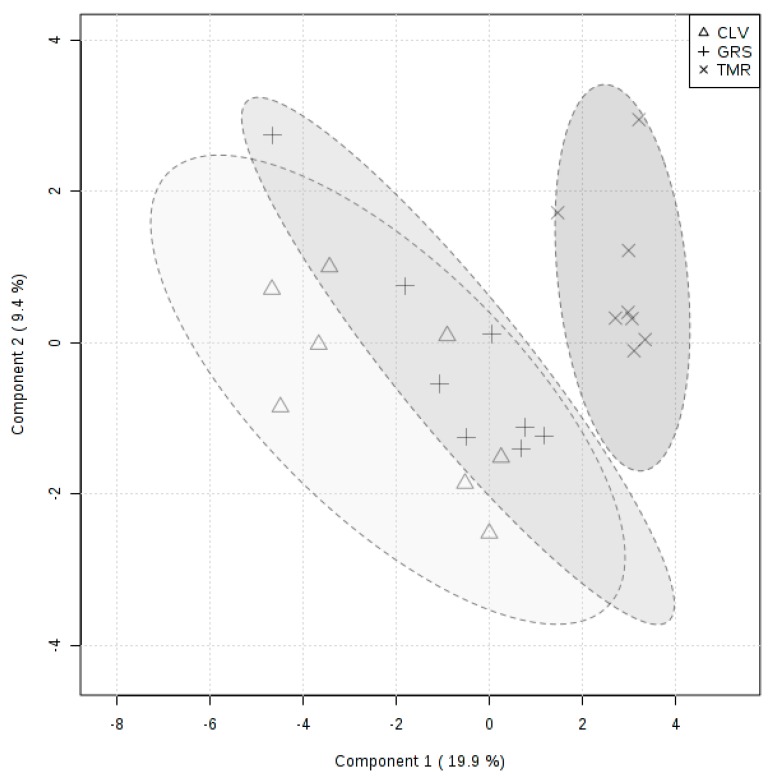
Partial-least-square discriminant analysis (PLS-DA) score plot for protein ingredient metabolome from milk of cows fed perennial ryegrass (GRS), perennial ryegrass/white clover (CLV), and total mixed ration (TMR) feeding systems, determined by LC–MS/MS.

**Figure 2:**
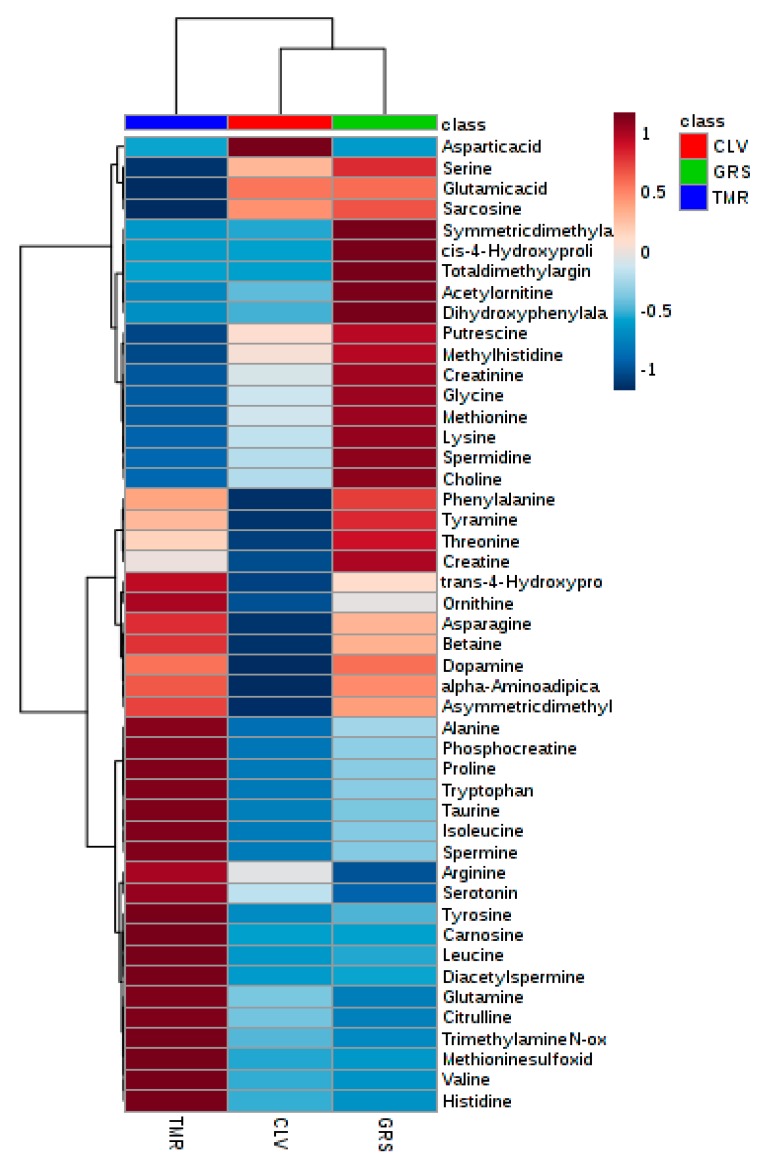
Heatmap showing average SMP and whey ingredient metabolites from cows fed on perennial ryegrass (GRS), perennial ryegrass/white clover (CLV), or total mixed ration (TMR) feeding systems, determined by LC–MS/MS. Degree of positive and negative correlation between metabolite and diet is indicated by +1 (red) to −1 (blue).

In the present study, the overall metabolome of the protein ingredients was shown to be significantly influenced by the type of feeding system when analyzed by principal component analysis (Supplementary Materials Figure S1) of the metabolomics analysis data. While both pasture-based systems show similar distributions, the distribution of the TMR system is narrow and distinct from both GRS and CLV, displaying low axial variance. Partial-least-square discriminant analysis (Figure 1) shows the significant overlap between the samples from the GRS and CLV systems and the pronounced separation between both of these systems and that of TMR. The overlap between the GRS and CLV samples is to be expected, given the similarity of these diets. This trend can also be shown clearly by using hierarchical clustering analysis (Figure 2), which represents the degree of positive or negative correlation of each metabolite to each feeding system. A clear separation between the TMR and pasture-based samples was also demonstrated by this analysis, offering evidence to support the applicability of LC–MS-based metabolomics for differentiation between dairy products derived from different feeding systems. This method may be appropriate for implementation into a milk-quality-determination laboratory setting, where the preparatory equipment used is typically readily available, and operators may already be trained in chromatographic and mass spectroscopy methods. However, in comparison to NMR, this method requires more extensive sample preparation (e.g., derivatization) and longer sample run times, though LC–MS offers greater sensitivity and greater potential for resolution of a larger number of compounds.

## 3. Materials and Methods

### 3.1. Materials

Raw milk was obtained from Teagasc Animal and Grassland Research and Innovation Centre (Moorepark, Fermoy, Co. Cork, Ireland). Hydrochloric acid and sodium hydroxide used for acid whey production were sourced from Sigma-Aldrich (Merck KGaA, Darmstadt, Germany).

### 3.2. Experimental Design

The experimental design for this study was the same as that previously described in studies which investigated the quality of butter [43], cheddar cheese [44], and raw milks [15,16] from these feeding systems. Briefly, fifty-four spring calving Friesian cows were randomly allocated to three groups (n = 18) at the Teagasc Animal and Grassland Research and Innovation Centre, Moorepark, Fermoy, Co. Cork, Ireland. Group 1 was housed indoors and fed a TMR diet, and Group 2 was maintained outdoors on perennial ryegrass only pasture (GRS), while Group 3 was also maintained outdoors on a perennial ryegrass/white clover pasture (CLV). For further information on the chemical and nutritional values of each of the diets see O’Callaghan et al. [16]. For further information on the allocation of pasture-based dry matter using estimates of pre-grazing herbage mass and daily post grazing sward heights, see Egan et al. [45]. Milk was collected from each of the groups in the trial for milk powder manufacture on two separate occasions over a two-week period in July 2017, to produce 2 independent batches of skim milk powder (SMP) from each feeding system at pilot plant scale. All of the milk powders within each batch were manufactured on the same day at Moorepark Technology Ltd. (Moorepark, Fermoy, Co. Cork, Ireland).

### 3.3. Ingredient Manufacture

#### 3.3.1. Skim Milk Preparation

Raw whole milk (approximately 1000 kg) was obtained from bulk milk tanks designated to each dietary treatment. This milk was preheated to 50 °C in an APV plate heat exchanger (SPX Flow Technology, Crawley, West Sussex, UK), followed by separation in a Westfalia centrifugal disk separator (GEA Westfalia, Oelde, Germany), and then it was pasteurized at 72 °C, for 15 s. The pasteurized skim milk was preheated to approximately 78 °C and concentrated to ~43% total solids (TS) in a Niro three-effect falling film evaporator (GEA Niro A/S, Soeborg, Denmark), at sequential effect temperatures of 73, 64, and 55 °C, respectively. The concentrate feed was then spray-dried, using a Niro Tall-Form Anhydro three-stage spray dryer (air inlet and outlet temperatures were set at 180 and 85 °C, respectively), using a high-pressure atomization system. External first and second fluid bed temperatures were set at 74 and 24 °C, respectively. All fines were returned from the cyclone to the second fluid bed, yielding a low-heat non-agglomerated skim milk powder (SMP) (~3% moisture).

#### 3.3.2. Sweet Whey Preparation

Raw whole milk from each dietary treatment was set aside from the bulk collection described in Section 3.3.1 and pasteurized at 72 °C, for 15 s, using a Microthermics^TM^ tubular heat-exchanger (Microthermics Inc., Raleigh, North Carolina, USA), and then stored in sterilized containers. Milk samples (~10 kg) were then added to a laboratory scale jacketed cheese-production vessel and preheated to 33 °C. Chymosin (Chy-Max Plus, 200 IMCU mL^−1^; Chr Hansen Ireland Ltd., Cork, Ireland) was diluted in 30 mL of deionized water and added to the milk (0.272 mL L^−1^), followed by controlled stirring for 3 min. The stirring paddles were then removed from the vessel and replaced with cutters. An aliquot (17 g) of the inoculated milk was weighed into a concentric cylinder in a Discovery HR-1 Hybrid Rheometer (TA Instruments, New Castle, Delaware, USA). After ~35 min, at an elastic modulus (*G*′) reading of 30 Pa, curd cutting was carried out for 1 h, at 45 °C. The whey was then separated from the curd, using cheesecloth, and stored at −80 °C. The whey was later filtered, using Whatman No. 1 filter paper, and clarified, using a 0.1 μm Sartocon Slice polyethersulfone cassette membrane (Sartorius AG, Göttigen, Germany), before being freeze-dried in a Labconco stoppering tray dryer equipped with a Freezone 12 plus vacuum collector/refrigerator unit (Labconco, Kansas City, Missouri, USA).

#### 3.3.3. Acid Whey Preparation

Skim milk powder from each dietary treatment was reconstituted to 9% TS and maintained at 20 °C in a water bath. Hydrochloric acid (HCl) (2 M) was added to the milk in order to decrease the pH to the isoelectric point of pH 4.6, and the precipitated casein curd was then removed from the whey, using cheesecloth. The pH of the whey was then readjusted to pH 6.7, using sodium hydroxide (NaOH), and it was later filtered, clarified, and freeze-dried, as described in Section 3.3.2.

#### 3.3.4. Ideal Whey Preparation

Skim milk powder from each dietary treatment was reconstituted to 9% TS and filtered through a 0.1 μm Sartocon Slice polyethersulfone cassette membrane, at approximately 2.0 bar under recirculation mode (30 °C) in order to separate micellar casein and whey protein. Both micellar casein from the retentate and the whey permeate stream were then freeze-dried, as described in Section 3.3.2.

### 3.4. Determination of Nitrogen Content

The total nitrogen content of each sample was determined through the Kjeldahl method, as described in ISO 8968-1 (2001), using a nitrogen-to-milk protein conversion factor of 6.38. Nonprotein nitrogen content was determined by precipitation of the protein component of each sample, using trichloroacetic acid (15% *w*/*v*). The precipitate was removed from the mixture by using Whatman No. 1 filter paper, and a Kjeldahl determination was then carried out on the filtrate, as described above.

### 3.5. Total Amino Acid Analysis

Acid hydrolysis was carried out to break complete proteins and peptides down to individual AAs. Proteins were hydrolyzed with 6 N HCl at 110 °C, for 23 h, using a Gals-Col combo mantle (Gals-Col, Terre Haute, USA) for the determination of all AAs except sulfur AAs and tryptophan. Methionine and cysteine were oxidized with performic acid to methionine sulfone and cysteic acid, respectively, and then hydrolyzed with HCl. The resulting hydrolysates were then diluted 1 in 2 with the internal standard, norleucine, to give a final concentration of 125 nm/mL. Amino acids were quantified by using a Jeol JLC-500/V amino acid analyzer (Jeol (UK) Ltd., Garden city, Herts, UK) fitted with a Jeol Na^+^ high-performance cation-exchange column. The analyzer uses an ion-exchange column with post-column online reactor derivatization with ninhydrin.

### 3.6. Liquid Chromatography–Mass Spectrometry (LC–MS)

Metabolite analysis was carried out at The Metabolomics Innovation Centre (University of Alberta, Edmonton, Alberta, Canada). A targeted quantitative metabolomics approach was used to analyze the milk samples, using a combination of direct injection mass spectrometry (DI-MS) with a reverse-phase LC–MS/MS assay. The method used combines the derivatization and extraction of analytes and the selective mass-spectrometric detection, using multiple reaction monitoring (MRM) pairs. Isotope-labeled internal standards were used for metabolite quantification. All the milk samples were thawed on ice and were vortexed and centrifuged at 13,000× *g*. Ten µL of each milk sample was loaded and dried in a stream of nitrogen, and 50 µL of a 5% solution of phenyl-isothiocyanate was then added for derivatization. After incubation, samples were dried again, using a nitrogen evaporator. Extraction of the metabolites was then achieved by adding 300 µL of methanol containing 5 mM of ammonium acetate. Extracts (150 µL) were diluted in a 1:1 ratio with water for LC–MS/MS analysis of AAs and biogenic amines. The remaining 150 µL of extracts was mixed with 400 µL of running solvent for DI-MS analysis of lipids and carnitines.

Mass spectrometric analysis was performed on a 4000 QTrap^®^ tandem mass spectrometry instrument (Applied Biosystems/MDS Analytical Technologies, Foster City, California, USA) equipped with an Agilent 1100 LC system. The samples were delivered to the mass spectrometer by an LC method, followed by a direct injection (DI) method. Data analysis was performed, and concentrations were calculated by using Analyst software 1.6.2.

### 3.7. Statistical Analysis

Statistical analysis was performed by using Genstat v18.1 (VSN International Ltd., Hemel Hempstead, Hertfordshire, UK). Datasets were analyzed for normality, using the Shapiro–Wilk’s test. Data were deemed normally distributed, and the analysis was carried out by using a 4 × 3 factorial ANOVA with post hoc Tukey test. The *p*-values < 0.05 were considered significant. Multivariate statistical analysis was carried out by using Metaboanalyst 3.0 [46] software, from which the figures were also generated.

## 4. Conclusions

The data presented in this study contribute to the overall characterization of the composition of milk products derived from the milk of cows fed via three widely practiced feeding systems. This lends an insight into the effect of these diets on the AA composition of milk, which has received limited attention to date. The significant differences observed in the total AA analysis suggest that the AA composition of milk may be more responsive to variation due to diet than previously assumed. Significant variation was observed in the average AA composition between each whey type (acid, sweet, and ideal), which may be an important consideration for nutritional formulations. The greater overall concentrations of free AAs in TMR-derived samples may be linked to the increased nonprotein nitrogen content of these milks. The significant effect of the type of feeding system on the metabolome of each ingredient type supports previous work examining the metabolome of pasture and concentrate-derived milk. As such, this implies that LC–MS-based metabolomics may be a suitable method for the differentiation of milks and whey products from different feeding systems.

## Figures and Tables

**Table 1:** Average total nitrogen and nonprotein nitrogen content of skim milk powder, rennet whey, ideal whey, and acid whey, as determined by Kjeldahl analysis.

Sample Type	Total Protein (% *w*/*w*)			Nonprotein Nitrogen (% *w*/*w*)		
	GRS	CLV	TMR	GRS	CLV	TMR
Skim milk powder	37.2 (±0.57) ^b^	37.5 (±0.06) ^d^	36.1 (±0.61) ^c^	0.27 (±0.01) ^b,A^	0.32 (±0.06) ^b,A,B^	0.37 (±0.00) ^c,B^
Sweet whey powder	9.27 (±0.29) ^a^	9.41 (±0.68) ^b,c^	9.64 (±0.17) ^b^	0.25 (±0.01) ^a,b^	0.27 (±0.04) ^a,b^	0.32 (±0.01) ^a,b,c^
Ideal whey powder	8.07 (±0.45) ^a^	6.98 (±0.61) ^a^	8.23 (±0.86) ^a,b^	0.21 (±0.01) ^a,b^	0.23 (±0.04) ^a^	0.28 (±0.03) ^a,b^
Acid whey powder	7.64 (±0.17) ^a^	7.98 (±0.17) ^a,b^	7.50 (±0.47) ^a^	0.19 (±0.01) ^a^	0.21 (±0.04) ^a^	0.25 (±0.01) ^a^

GRS—cows fed perennial ryegrass only. CLV—cows fed perennial ryegrass/20% white clover sward. TMR—cows fed indoor total mixed ration *ad-libitum*. ^a,b,c,d^ indicates values within a column not sharing a common lower-case superscript letter differed significantly (*p* < 0.05). ^A,B^ indicates values within a row not sharing a common upper-case superscript letter differed significantly (*p* < 0.05).

**Table 2:** Total amino acid composition of skim milk powder, sweet whey, ideal whey, and acid whey, determined by high-performance cation exchange.

TAA g/kg Total Protein	Skim Milk Powder			Sweet Whey Powder			Ideal Whey Powder			Acid Whey Powder		
	GRS	CLV	TMR	GRS	CLV	TMR	GRS	CLV	TMR	GRS	CLV	TMR
Cysteic acid	11.2	11.8	10.6	29.4	29.0	28.9	34.1	31.4	32.9	34.6	32.9	34.0
Taurine	9.52	8.97	9.78	32.4	36.2	35.7	38.3	50.8	35.5	35.7	41.7	41.4
Methionine Sulfone	33.3	34.3	34.6	21.0	21.3	20.0	23.8	21.0	21.2	20.0	20.4	20.3
Asparagine	73.3	73.9	74.1	97.0	96.4	94.0	99.8	93.7	96.9	101	98.1	98.6
Threonine	41.1	41.0	41.2	62.0	60.4	57.5	41.5	37.7	40.1	41.4	40.2	39.2
Serine	50.1	49.9	49.9	41.2	39.7	37.8	35.4	31.0	33.8	34.6	33.1	32.1
Glutamine	190	193	189	159	155	142	155	138	143	159	153	139
Glycine	16.3	16.7	15.6	20.2 ^b^	18.9 ^b^	15.8 ^a^	20.6	20.0	18.2	19.6 ^b^	18.6 ^a,b^	16.4 ^a^
Alanine	28.4	28.0	27.9	39.6	41.2	36.9	38.6	32.0	31.4	38.0	35.6	34.8
Cysteine	7.13	8.14	7.88	20.7	24.4	21.5	25.7 ^a,b^	34.6 ^b^	21.6 ^a^	22.2 ^a,b^	30.4 ^b^	15.4 ^a^
Valine	55.3	57.8	56.2	54.6	52.9	52.0	51.8	46.6	43.3	51.3 ^b^	48.9 ^a,b^	36.7 ^a^
Isoleucine	43.7	45.6	44.5	53.8	51.5	48.4	44.1	38.1	40.9	46.6	44.0	42.7
Leucine	93.5	95.5	94.7	88.1	88.4	82.1	98.5	91.5	92.5	103	100	93.7
Tyrosine	30.2	34.1	32.7	8.92	9.63	12.0	13.7	12.5	13.2	8.87	10.5	8.77
Phenylalanine	42.4	42.2	40.8	24.3	22.8	23.2	31.1 ^b^	24.5 ^a^	28.4 ^a,b^	30.0	30.0	26.9
Histidine	35.7	35.4	35.7	25.3	28.2	25.6	33.6	35.7	31.7	40.2	41.2	35.7
Lysine	72.6	73.1	73.7	75.7	74.5	70.8	77.8	68.4	71.5	82.0	77.8	74.7
Arginine	31.4	32.0	31.5	19.8	19.5	20.0	24.1	23.3	24.9	25.3	24.2	23.0
Proline	84.2	86.1	88.7	44.0	48.0	35.0	27.2	24.2	32.9	30.3	25.1	21.9

GRS—cows fed perennial ryegrass only. CLV—cows fed perennial ryegrass/20% white clover sward. TMR—cows fed indoor total mixed ration *ad-libitum*. ^a,b^ indicates values within a row for each ingredient not sharing a common superscript letter differed significantly (*p* < 0.05).

**Table 3:** Average values for total amino acid composition of sweet whey, ideal whey, and acid whey, determined by high-performance cation exchange.

TAA g/kg Total Protein	Sweet Whey Powder	Ideal Whey Powder	Acid Whey Powder
Cysteic acid	29.1 (±0.26) ^a^	32.8 (±1.34) ^b^	33.8 (±0.89) ^b^
Taurine	34.8 (±2.06)	41.5 (±8.16)	39.6 (±3.39)
Methionine Sulfone	20.8 (±0.65)	22.0 (±1.55)	20.2 (±0.24)
Asparagine	95.8 (±1.57)	96.8 (±3.04)	99.3 (±1.69)
Threonine	60.0 (±2.32) ^b^	39.8 (±1.94) ^a^	40.3 (±1.08) ^a^
Serine	39.6 (±1.67) ^b^	33.4 (±2.22) ^a^	33.3 (±1.24) ^a^
Glutamine	152 (±8.50)	145 (±8.61)	150 (±10.2)
Glycine	18.3 (±2.30) ^a^	19.6 (±1.23) ^b^	18.2 (±1.65) ^a^
Alanine	39.2 (±2.13) ^b^	34.0 (±3.97) ^a^	36.1 (±1.63) ^a,b^
Cysteine	22.2 (±1.98) ^a^	27.3 (±6.61) ^b^	22.7 (±7.52) ^a^
Valine	53.2 (±1.36) ^b^	47.2 (±4.30) ^a^	45.7 (±7.83) ^a^
Isoleucine	51.2 (±2.72) ^b^	41.0 (±3.02) ^a^	44.5 (±1.98) ^a^
Leucine	86.2 (±3.57) ^a^	94.2 (±3.77) ^b^	99.4 (±5.23) ^b^
Tyrosine	10.2 (±1.63) ^a^	13.2 (±0.64) ^b^	9.38 (±0.97) ^a^
Phenylalanine	23.4 (±0.77) ^a^	28.0 (±3.35) ^b^	29.0 (±1.80) ^b^
Histidine	26.4 (±1.57) ^a^	33.7 (±1.98) ^b^	39.0 (±2.96) ^c^
Lysine	73.7 (±2.51)	72.6 (±4.81)	78.2 (±3.63)
Arginine	19.8 (±0.26) ^a^	24.1 (±0.82) ^b^	24.2 (±1.13) ^b^
Proline	42.3 (±6.69) ^b^	28.1 (±4.42) ^a^	25.8 (±4.25) ^a^

GRS—cows fed perennial ryegrass only. CLV—cows fed perennial ryegrass/20% white clover sward. TMR—cows fed indoor total mixed ration *ad-libitum*. ^a,b,c^ indicates values within a row not sharing a common superscript letter differed significantly (*p* < 0.05).

**Table 4:** Average concentrations (μM) of metabolites which showed significant differences between feeding systems for skim milk powder, sweet whey, ideal whey, and acid whey, determined by LC–MS/MS.

Ingredient	Metabolite (μM)	GRS	CLV	TMR
Skim milk powder	Glutamine	4.38 (±0.54) ^a^	4.95 (±1.24) ^a^	12.4 (±0.28) ^b^
	Phosphocreatine	8.05 (±1.70) ^a,b^	6.32 (±0.56) ^a^	16.3 (±4.45) ^b^
	Serine	22.1 (±0.78) ^b^	18.3 (±1.20) ^a,b^	10.0 (±0.55) ^a^
	Valine	7.49 (±0.12) ^a^	7.27 (±1.09) ^a^	11.3 (±1.34) ^b^
Sweet whey powder	Glutamine	1.36 *^a^	0.187 (±0.02) ^a^	7.02 (±3.20) ^b^
	Phosphocreatine	9.65 (±0.93) ^a^	6.49 (±0.70) ^a^	22.6 (±6.01) ^b^
	Serine	25.3 (±3.11) ^b^	18.7 (±2.33) ^a,b^	9.31 (±0.64) ^a^
Ideal whey powder	Glutamine	4.04 (±0.23) ^a^	3.19 (±1.77) ^a^	11.5 (±2.64) ^b^
	Phosphocreatine	7.21 (±1.64) ^a^	5.37 (±0.61) ^a^	17.1 (±1.41) ^b^
	Serine	21.9 (±1.13) ^b^	18.8 (±1.27) ^a,b^	9.62 (±1.10) ^a^
	Valine	7.00 (±0.74) ^a^	7.92 (±1.43) ^a,b^	10.6 (±0.07) ^b^
Acid whey powder	Glutamine	2.39 (±1.74) ^a^	2.97 (±1.07) ^a^	12.3 (±0.42) ^b^
	Valine	6.80 (±1.97) ^a^	7.29 (±0.69) ^a^	12.0 (±1.63) ^b^

GRS—cows fed perennial ryegrass only. CLV—cows fed perennial ryegrass/20% white clover sward. TMR—cows fed indoor total mixed ration *ad-libitum*. ^a,b^ indicates values within a row not sharing a common superscript letter differed significantly (*p* < 0.05). * Denotes where a replicate was below the limit of detection or limit of quantification.

## Supplementary Materials

The following are available online at https://www.mdpi.com/2218-1989/9/12/305/s1, Table S1: Average concentrations of total metabolites (μM) for skim milk powder derived from perennial ryegrass (GRS), perennial ryegrass/white clover (CLV) and total mixed ration (TMR) feeding systems, determined by LC-MS/MS., Table S2: Average concentrations of total metabolites (μM) for sweet whey powder derived from perennial ryegrass (GRS), perennial ryegrass/white clover (CLV) and total mixed ration (TMR) feeding systems, determined by LC-MS/MS., Table S3: Average concentrations of total metabolites (μM) for ideal whey powder derived from perennial ryegrass (GRS), perennial ryegrass/white clover (CLV) and total mixed ration (TMR) feeding systems, determined by LC-MS/MS., Table S4: Average concentrations of total metabolites (μM) for acid whey powder derived from perennial ryegrass (GRS), perennial ryegrass/white clover (CLV) and total mixed ration (TMR) feeding systems, determined by LC-MS/MS., Table S5: Average concentrations of total metabolites (μM) from skim milk powder, sweet whey powder, ideal whey powder and acid whey powder derived from perennial ryegrass (GRS), perennial ryegrass/white clover (CLV) and total mixed ration (TMR) feeding systems, determined by LC-MS/MS., Figure S1: Principal component analysis (PCA) score plot for metabolomics analysis of protein ingredients from perennial ryegrass (GRS), perennial ryegrass/white clover (CLV) and total mixed ration (TMR) feeding systems, determined by LC-MS/MS.
