# Supplementary material for: Impact of Bovine Diet on Metabolomic Profile of Skim Milk and Whey Protein Ingredients
Source: Metabolites. 2019 Dec 17;9(12):305. doi: 10.3390/metabo9120305 (PMC6950411; doi:10.3390/metabo9120305)
Supplement: Supplementary file 1 [file metabolites-09-00305-s001.pdf]

Supplementary data

Table S1: Average concentrations of total metabolites (μM) for skim milk powder derived from perennial ryegrass (GRS), perennial ryegrass/white clover (CLV) and total mixed ration (TMR) feeding systems, determined by LC-MS/MS.
[a,b] indicates values within a row not sharing a common superscript letter differed significantly (p < 0.05).
* denotes where a replicate was below the limit of detection or limit of quantification.

Metabolite (μM)	GRS	CLV	TMR
Acetylornitine	0.97 (± 0.07)	0.60 (± 0.08)	0.88 (± 0.54)
Alanine	34.1 (± 2.90)	30.2 (± 3.32)	41.9 (± 1.20)
alpha-Aminoadipic acid	6.86 (± 0.47)	5.45 (± 2.26)	7.42 (± 1.83)
Arginine	14.2 (± 0.42)	14.6 (± 1.70)	16.6 (± 0.14)
Asparagine	1.41 (± 0.84)	2.11*	1.29 (± 0.16)
Aspartic acid	10.2 (± 3.34)	17.3 (± 4.38)	11.5 (± 2.38)
Asymmetric dimethylarginine	0.09 (± 0.09)	0.05 (± 0.03)	0.09 (± 0.01)
Betaine	74.2 (± 20.01)	61.0 (± 19.30)	94.2 (± 12.52)
Carnosine	0.35 (± 0.02)	0.40 (± 0.04)	0.47 (± 0.08)
Choline	1245 (± 190.92)	1024 (± 319.61)	1035 (± 35.36)
cis-4-Hydroxyproline	0.31 (± 0.00)	0.34 (± 0.01)	0.32 (± 0.01)
Citrulline	1.89 (± 0.16)	1.71 (± 0.94)	2.29 (± 0.47)
Creatine	512 (± 7.07)	507 (± 4.24)	567 (± 65.76)
Creatinine	111 (± 2.83)	93.1 (± 14.00)	92.6 (± 3.82)
Diacetylspermine	0.03 (± 0.00)	0.03 (± 0.00)	0.03 (± 0.00)
Dihydroxyphenylalanine	0.18 (± 0.02)	0.16 (± 0.00)	0.15 (± 0.02)
Dopamine	0.08 (± 0.00)	0.08 (± 0.00)	0.08 (± 0.00)
Glutamic acid	256(± 7.07)	284 (± 12.73)	241 (± 7.07)
Glutamine	4.38 (± 0.54)^{a}	4.95 (± 1.24)^{a}	12.4 (± 0.28)^{b}
Glycine	65.0 (± 28.35)	54.3 (± 13.86)	74.1 (± 16.55)
Histidine	2.35 (± 0.57)	2.37 (± 0.42)	3.41 (± 0.43)
Isoleucine	2.49 (± 0.62)	2.40 (± 0.03)	4.32 (± 0.23)
Leucine	2.67 (± 1.14)	3.10 (± 0.95)	5.59 (± 1.20)
Lysine	15.8 (± 2.05)	13.8 (± 1.48)	12.9 (± 0.28)
Methionine	0.66 (± 0.03)	0.71 (± 0.18)	0.42 (± 0.08)
Methionine sulfoxide	0.07*	0.00*	0.22 (± 0.13)
Methylhistidine	1.20 (± 0.15)	0.99 (± 0.07)	1.00 (± 0.07)
Ornithine	2.24 (± 0.68)	2.11 (± 0.87)	4.14 (± 0.57)
Phenylalanine	1.44 (± 0.01)	1.19 (± 0.06)	1.42 (± 0.00)
Phosphocreatine	8.05 (± 1.70)^{a,b}	6.32 (± 0.56)^{a}	16.3 (± 4.45)^{b}
Proline	15.3 (± 0.71)	14.0 (± 0.64)	19.1 (± 1.41)
Putrescine	0.07 (± 0.00)	0.08 (± 0.01)	0.04 (± 0.00)
Sarcosine	0.48 (± 0.05)	0.56 (± 0.03)	0.42 (± 0.09)
Serine	22.1 (± 0.78)^{b}	18.3 (± 1.20)^{a,b}	10.0 (± 0.55)^{a}
Serotonin	0.01 (± 0.00)	0.01 (± 0.00)	0.01 (± 0.00)
Spermidine	0.83 (± 0.13)	0.77 (± 0.06)	0.82 (± 0.03)

Spermine	0.48 (± 0.05)	0.50 (± 0.13)	0.59 (± 0.02)
Symmetric dimethylarginine	4.36 (± 0.28)	3.26 (± 0.24)	3.38 (± 0.87)
Taurine	26.5 (± 2.69)	25.0 (± 1.84)	30.3 (± 1.84)
Threonine	4.49 (± 0.61)	3.82 (± 0.35)	4.99 (± 0.67)
Total dimethylarginine	4.45 (± 0.19)	3.31 (± 0.21)	3.47 (± 0.86)
trans-4-Hydroxyproline	4.39 (± 0.04)	4.25 (± 0.15)	4.53 (± 0.22)
Trimethylamine N-oxide	3.35 (± 0.69)	2.83 (± 0.08)	3.22 (± 0.02)
Tryptophan	0.83 (± 0.02)	0.74 (± 0.06)	0.83 (± 0.11)
Tyramine	0.01*	0.01 (± 0.01)	0.00 (± 0.00)
Tyrosine	0.31 (± 0.01)	0.28 (± 0.08)	0.64 (± 0.02)
Valine	7.49 (± 0.12)[a]	7.27 (± 1.09)[a]	11.3 (± 1.34)[b]

Table S2: Average concentrations of total metabolites (μM) for sweet whey powder derived from perennial ryegrass (GRS), perennial ryegrass/white clover (CLV) and total mixed ration (TMR) feeding systems, determined by LC-MS/MS.

[a,b] indicates values within a row not sharing a common superscript letter differed significantly ($p < 0.05$).

* denotes where a replicate was below the limit of detection or limit of quantification.

**Metabolite (μM)**	**GRS**	**CLV**	**TMR**
Acetylornitine	1.26 (± 0.25)	0.87 (± 0.15)	0.80 (± 0.04)
Alanine	44.6 (± 7.64)	35.2 (± 2.90)	39.2 (± 5.30)
alpha-Aminoadipic acid	10.6 (± 3.15)	9.32 (± 2.67)	7.69 (± 1.15)
Arginine	16.0 (± 2.90)	14.7 (± 0.00)	14.9 (± 0.64)
Asparagine	1.25*	1.70*	3.24 (± 1.13)
Aspartic acid	10.5 (± 0.28)	18.7 (± 11.31)	10.2 (± 0.66)
Asymmetric dimethylarginine	0.18 (± 0.06)	0.04 (± 0.02)	0.06 (± 0.04)
Betaine	80.7 (± 16.48)	63.9 (± 23.05)	73.0 (± 19.09)
Carnosine	0.40 (± 0.06)	0.39 (± 0.04)	0.48 (± 0.08)
Choline	1415 (± 219.20)	1224 (± 574.17)	892 (± 1.41)
cis-4-Hydroxyproline	0.34 (± 0.00)	0.33 (± 0.01)	0.32 (± 0.01)
Citrulline	1.50 (± 0.28)	1.38 (± 1.20)	2.46 (± 0.69)
Creatine	761 (± 108.19)	543 (± 26.16)	561 (± 101.82)
Creatinine	115 (± 14.85)	93.6 (± 6.93)	72.9 (± 5.59)
Diacetylspermine	0.03 (± 0.00)	0.03 (± 0.00)	0.03 (± 0.00)
Dihydroxyphenylalanine	0.21 (± 0.02)	0.17 (± 0.02)	0.17 (± 0.00)
Dopamine	0.08 (± 0.00)	0.08*	0.08 (± 0.00)
Glutamic acid	318 (± 46.67)	297 (± 33.23)	238 (± 9.19)
Glutamine	1.36*[a]	0.187 (± 0.02)[a]	7.02 (± 3.20)[b]
Glycine	93.9 (± 55.37)	61.7 (± 1.13)	47.8 (± 11.17)
Histidine	2.61 (± 0.74)	2.50 (± 0.16)	3.11 (± 0.53)
Isoleucine	3.10 (± 0.71)	2.49 (± 0.39)	3.81 (± 0.28)
Leucine	3.20 (± 0.48)	3.24 (± 0.24)	4.16 (± 2.14)
Lysine	18.6 (± 2.90)	14.0 (± 2.12)	12.5 (± 1.13)
Methionine	1.34 (± 0.08)	1.23 (± 0.04)	0.95 (± 0.14)
Methionine sulfoxide	0.42 (± 0.14)	0.47 (± 0.06)	0.25 (± 0.31)
Methylhistidine	1.26 (± 0.25)	1.13 (± 0.28)	0.92 (± 0.04)
Ornithine	2.89 (± 1.20)	1.92 (± 0.03)	2.76 (± 0.28)
Phenylalanine	1.72 (± 0.08)	1.51 (± 0.06)	1.46 (± 0.02)
Phosphocreatine	9.65 (± 0.93)[a]	6.49 (± 0.70)[a]	22.6 (± 6.01)[b]
Proline	16.8 (± 2.33)	15.5 (± 0.07)	18.3 (± 0.49)
Putrescine	0.06 (± 0.01)	0.06 (± 0.02)	0.04 (± 0.04)
Sarcosine	0.54 (± 0.21)	0.47 (± 0.17)	0.33 (± 0.03)
Serine	25.3 (± 3.11)[b]	18.7 (± 2.33)[a,b]	9.31 (± 0.64)[a]
Serotonin	0.01 (± 0.00)	0.01 (± 0.00)	0.01 (± 0.00)
Spermidine	0.34 (± 0.04)	0.35 (± 0.04)	0.30 (± 0.00)
Spermine	0.05 (± 0.00)	0.06 (± 0.03)	0.08 (± 0.01)
Symmetric	4.60 (± 0.95)	3.30 (± 0.63)	3.06 (± 0.39)

dimethylarginine			
Taurine	29.8 (± 6.36)	28.4 (± 0.49)	30.5 (± 1.06)
Threonine	5.19 (± 1.20)	3.41 (± 0.50)	4.41 (± 0.18)
Total dimethylarginine	4.79 (± 1.01)	3.35 (± 0.64)	3.12 (± 0.43)
trans-4-Hydroxyproline	4.84 (± 0.30)	4.45 (± 0.17)	4.41 (± 0.37)
Trimethylamine N-oxide	3.33 (± 0.41)	3.19 (± 0.01)	3.32 (± 0.35)
Tryptophan	0.89 (± 0.35)	0.79 (± 0.01)	0.70 (± 0.10)
Tyramine	0.01 (± 0.00)	0.01 (± 0.00)	0.01 (± 0.00)
Tyrosine	0.42 (± 0.26)	0.28 (± 0.02)	0.51 (± 0.22)
Valine	8.50 (± 2.83)	7.78 (± 0.72)	10.6 (± 0.00)

Table S3: Average concentrations of total metabolites (μM) for ideal whey powder derived from perennial ryegrass (GRS), perennial ryegrass/white clover (CLV) and total mixed ration (TMR) feeding systems, determined by LC-MS/MS.
[a,b] indicates values within a row not sharing a common superscript letter differed significantly ($p < 0.05$).
* denotes where a replicate was below the limit of detection or limit of quantification.

Metabolite (μM)	GRS	CLV	TMR
Acetylornitine	0.96 (± 0.30)	0.98 (± 0.22)	0.78 (± 0.07)
Alanine	36.9 (± 4.03)	33.0 (± 1.84)	42.3 (± 4.03)
alpha-Aminoadipic acid	7.56 (± 1.16)	6.01 (± 2.51)	6.00 (± 0.57)
Arginine	13.4 (± 1.20)	13.8 (± 0.21)	15.6 (± 0.21)
Asparagine	1.45 (± 0.37)	0.34*	3.18 (± 0.95)
Aspartic acid	11.6 (± 0.99)	10.8 (± 2.93)	15.2 (± 2.19)
Asymmetric dimethylarginine	0.07 (± 0.03)	0.06 (± 0.02)	0.15 (± 0.01)
Betaine	85.5 (± 17.96)	66.8 (± 9.90)	83.8 (± 25.81)
Carnosine	0.38 (± 0.04)	0.37 (± 0.00)	0.43 (± 0.01)
Choline	1225 (± 120.21)	1020 (± 282.84)	906 (± 19.80)
cis-4-Hydroxyproline	0.33 (± 0.01)	0.31 (± 0.01)	0.32 (± 0.01)
Citrulline	1.40 (± 0.25)	1.67 (± 0.13)	2.66 (± 0.16)
Creatine	513 (± 29.70)	519 (± 12.02)	500 (± 55.86)
Creatinine	125 (± 12.02)	107 (± 17.47)	101 (± 8.84)
Diacetylspermine	0.03 (± 0.00)	0.03 (± 0.00)	0.03 (± 0.00)
Dihydroxyphenylalanine	0.18 (± 0.01)	0.18 (± 0.01)	0.15 (± 0.00)
Dopamine	0.08 (± 0.00)	0.08 (± 0.00)	0.08 (± 0.00)
Glutamic acid	266 (± 27.58)	269 (± 70.00)	282 (± 26.87)
Glutamine	4.04 (± 0.23)[a]	3.19 (± 1.77)[a]	11.5 (± 2.64)[b]
Glycine	66.8 (± 15.49)	61.2 (± 5.37)	56.4 (± 7.85)
Histidine	2.35 (± 0.50)	2.39 (± 0.45)	3.02 (± 0.17)
Isoleucine	2.76 (± 0.08)	2.78 (± 0.44)	3.86 (± 0.38)
Leucine	2.56 (± 1.63)	2.68 (± 1.61)	5.54 (± 0.10)
Lysine	15.8 (± 1.20)	12.8 (± 0.64)	13.2 (± 2.33)
Methionine	0.41 (± 0.29)	0.32 (± 0.37)	0.31 (± 0.07)
Methionine sulfoxide	0.20 (± 0.09)	0.29 (± 0.07)	0.35 (± 0.04)
Methylhistidine	1.26 (± 0.13)	0.92 (± 0.11)	0.83 (± 0.03)
Ornithine	2.54 (± 0.64)	2.37 (± 1.01)	2.79 (± 0.75)
Phenylalanine	1.46 (± 0.08)	1.38 (± 0.06)	1.38 (± 0.01)
Phosphocreatine	7.21 (± 1.64)[a]	5.37 (± 0.61)[a]	17.1 (± 1.41)[b]
Proline	15.8 (± 0.07)	14.7 (± 1.27)	19.0 (± 1.13)
Putrescine	0.05 (± 0.01)	0.05 (± 0.04)	0.02 (± 0.02)
Sarcosine	0.56 (± 0.12)	0.51 (± 0.04)	0.48 (± 0.05)
Serine	21.9 (± 1.13)[b]	18.8 (± 1.27)[a,b]	9.62 (± 1.10)[a]
Serotonin	0.01 (± 0.00)	0.01 (± 0.00)	0.01 (± 0.00)
Spermidine	0.29 (± 0.04)	0.26 (± 0.05)	0.24 (± 0.06)
Spermine	0.07 (± 0.01)	0.07 (± 0.01)	0.07 (± 0.00)
Symmetric	3.82 (± 0.08)	2.80 (± 0.46)	2.73 (± 0.25)

dimethylarginine			
Taurine	26.9 (± 2.33)	26.4 (± 1.56)	29.6 (± 1.70)
Threonine	4.88 (± 0.09)	3.90 (± 0.55)	4.08 (± 1.00)
Total dimethylarginine	3.90 (± 0.05)	2.86 (± 0.48)	2.88 (± 0.26)
trans-4-Hydroxyproline	4.21 (± 0.40)	4.03 (± 0.76)	4.53 (± 0.07)
Trimethylamine N-oxide	2.77 (± 0.30)	2.82 (± 0.04)	3.10 (± 0.29)
Tryptophan	0.70 (± 0.06)	0.64 (± 0.11)	0.75 (± 0.02)
Tyramine	0.01 (± 0.00)	0.00*	0.01 (± 0.01)
Tyrosine	0.27 (± 0.04)	0.28 (± 0.16)	0.48 (± 0.18)
Valine	7.00 (± 0.74)[a]	7.92 (± 1.43)[a,b]	10.6 (± 0.07)[b]

Table S4: Average concentrations of total metabolites (μM) for acid whey powder derived from perennial ryegrass (GRS), perennial ryegrass/white clover (CLV) and total mixed ration (TMR) feeding systems, determined by LC-MS/MS.

[a,b] indicates values within a row not sharing a common superscript letter differed significantly ($p < 0.05$).

* denotes where a replicate was below the limit of detection or limit of quantification.

**Metabolite (μM)**	**GRS**	**CLV**	**TMR**
Acetylornitine	4.18 (± 0.16)	2.58 (± 0.11)	2.36 (± 1.53)
Alanine	27.7 (± 2.19)	34.1 (± 5.37)	40.6 (± 3.04)
alpha-Aminoadipic acid	23.9 (± 7.14)	28.0 (± 28.61)	40.8 (± 2.26)
Arginine	12.9 (± 1.63)	16.4 (± 3.11)	16.0 (± 1.13)
Asparagine	2.98 (± 0.16)	0.32*	1.32 (± 0.12)
Aspartic acid	6.98 (± 1.03)	13.3 (± 12.47)	4.95 (± 0.34)
Asymmetric dimethylarginine	0.06 (± 0.01)	0.07 (± 0.00)	0.11 (± 0.04)
Betaine	75.9 (± 26.87)	70.7 (± 37.19)	84.9 (± 6.43)
Carnosine	0.33 (± 0.09)	0.30 (± 0.06)	0.39 (± 0.01)
Choline	1160 (± 98.99)	1076 (± 501.34)	950 (± 84.85)
cis-4-Hydroxyproline	0.31 (± 0.00)	0.31*	0.33*
Citrulline	0.33 (± 0.13)	0.66 (± 0.11)	0.86 (± 0.32)
Creatine	483 (± 60.10)	661 (± 119.50)	612 (± 144.96)
Creatinine	99.4 (± 3.68)	104 (± 14.35)	94.0 (± 5.52)
Diacetylspermine	0.03 (± 0.00)	0.03 (± 0.00)	0.03 (± 0.00)
Dihydroxyphenylalanine	0.14 (± 0.01)	0.12 (± 0.01)	0.15 (± 0.05)
Dopamine	0.08 (± 0.00)	0.08 (± 0.00)	0.08 (± 0.00)
Glutamic acid	262 (± 37.48)	255 (± 69.30)	234 (± 25.46)
Glutamine	2.39 (± 1.74)[a]	2.97 (± 1.07)[a]	12.3 (± 0.42)[b]
Glycine	56.1 (± 9.62)	57.3 (± 0.28)	41.2 (± 0.49)
Histidine	2.34 (± 0.55)	2.49 (± 0.01)	3.21 (± 0.15)
Isoleucine	2.80 (± 0.02)	2.58 (± 1.53)	3.73 (± 1.13)
Leucine	12.0 (± 5.82)	8.28 (± 3.57)	14.6 (± 0.71)
Lysine	12.7 (± 4.04)	12.5 (± 1.13)	11.4 (± 7.95)
Methionine	0.47 (± 0.10)	0.49 (± 0.10)	0.43 (± 0.24)
Methionine sulfoxide	0.16 (± 0.21)	0.44 (± 0.25)	0.14 (± 0.08)
Methylhistidine	1.27 (± 0.14)	1.36 (± 0.13)	0.97 (± 0.16)
Ornithine	3.22 (± 0.83)	2.68 (± 0.04)	3.35 (± 0.82)
Phenylalanine	1.22 (± 0.09)	1.09 (± 0.21)	1.41 (± 0.29)
Phosphocreatine	3.56 (± 0.24)	3.66 (± 0.84)	4.35 (± 0.47)
Proline	14.8 (± 2.47)	14.7 (± 2.12)	19.1 (± 2.26)
Putrescine	0.08 (± 0.02)	0.05*	0.01*
Sarcosine	0.41 (± 0.06)	0.39 (± 0.01)	0.40 (± 0.01)
Serine	20.2 (± 0.64)	17.5 (± 8.27)	11.2 (± 2.63)
Serotonin	0.01 (± 0.00)	0.01 (± 0.00)	0.01 (± 0.00)
Spermidine	0.71 (± 0.04)	0.58 (± 0.15)	0.56 (± 0.12)
Spermine	0.26 (± 0.07)	0.22 (± 0.08)	0.30 (± 0.21)
Symmetric	3.40 (± 0.38)	2.94 (± 1.58)	2.77 (± 0.03)

dimethylarginine			
Taurine	26.0 (± 5.16)	26.0 (± 3.04)	30.0 (± 3.46)
Threonine	1.69 (± 0.76)	1.02 (± 0.40)	1.28 (± 0.41)
Total dimethylarginine	3.46 (± 0.37)	3.01 (± 1.58)	2.88 (± 0.07)
trans-4-Hydroxyproline	4.09 (± 1.12)	4.01 (± 0.11)	4.48 (± 0.38)
Trimethylamine N-oxide	2.57 (± 0.05)	3.21 (± 0.69)	3.13 (± 0.48)
Tryptophan	0.45 (± 0.11)	0.57 (± 0.10)	0.58 (± 0.12)
Tyramine	0.01 (± 0.00)	0.00 (± 0.00)	0.00*
Tyrosine	0.00*	0.00*	0.08*
Valine	6.80 (± 1.97)[a]	7.29 (± 0.69)[a]	12.0 (± 1.63)[b]

Table S5: Average concentrations of total metabolites (µM) from skim milk powder, sweet whey powder, ideal whey powder and acid whey powder derived from perennial ryegrass (GRS), perennial ryegrass/white clover (CLV) and total mixed ration (TMR) feeding systems, determined by LC-MS/MS.

[a,b] indicates values within a row not sharing a common superscript letter differed significantly ($p < 0.05$).

Metabolite (µM)	GRS	CLV	TMR
Acetylornitine	1.84 (± 1.57)	1.26 (± 0.90)	1.20 (± 0.77)
Alanine	35.8 (± 7.02)[a,b]	33.1 (± 2.15)[a]	41.0 (± 1.40)[b]
alpha-Aminoadipic acid	12.2 (± 7.93)	12.2 (± 10.66)	15.5 (± 16.90)
Arginine	14.1 (± 1.36)	14.9 (± 1.11)	15.8 (± 0.74)
Asparagine	1.77 (± 0.81)[a,b]	1.12 (± 0.92)[a]	2.26 (± 1.10)[b]
Aspartic acid	9.83 (± 1.99)	15.0 (± 3.62)	10.5 (± 4.22)
Asymmetric dimethylarginine	0.10 (± 0.05)	0.06 (± 0.01)	0.10 (± 0.04)
Betaine	79.1 (± 5.10)[a,b]	65.6 (± 4.16)[a]	83.9 (± 8.66)[b]
Carnosine	0.36 (± 0.03)[a]	0.36 (± 0.05)[a]	0.44 (± 0.04)[b]
Choline	1261 (± 108.73)[b]	1085 (± 95.49)[a,b]	945 (± 64.43)[a]
cis-4-Hydroxyproline	0.32 (± 0.01)	0.32 (± 0.01)	0.32 (± 0.01)
Citrulline	1.28 (± 0.67)[a]	1.35 (± 0.49)[a]	2.07 (± 0.82)[b]
Creatine	567 (± 130.27)	557 (± 70.49)	559 (± 46.08)
Creatinine	112 (± 10.36)[b]	99.3 (± 6.97)[a,b]	90.1 (± 11.96)[a]
Diacetylspermine	0.03 (± 0.00)	0.03 (± 0.00)	0.03 (± 0.00)
Dihydroxyphenylalanine	0.18 (± 0.03)	0.16 (± 0.03)	0.16 (± 0.01)
Dopamine	0.08 (± 0.00)	0.08 (± 0.00)	0.08 (± 0.00)
Glutamic acid	275 (± 28.76)	276 (± 18.09)	248 (± 22.43)
Glutamine	3.04 (± 1.42)[a]	2.82 (± 1.97)[a]	10.8 (± 2.56)[b]
Glycine	70.4 (± 16.30)	58.6 (± 3.49)	54.9 (± 14.26)
Histidine	2.41 (± 0.13)[a]	2.44 (± 0.07)[a]	3.18 (± 0.17)[b]
Isoleucine	2.78 (± 0.25)[a]	2.56 (± 0.16)[a]	3.93 (± 0.27)[b]
Leucine	5.10 (± 4.60)[a,b]	4.32 (± 2.65)[a]	7.47 (± 4.80)[b]
Lysine	15.7 (± 2.37)	13.3 (± 0.74)	12.5 (± 0.78)
Methionine	0.72 (± 0.42)	0.69 (± 0.40)	0.53 (± 0.29)
Methionine sulfoxide	0.21 (± 0.15)	0.40 (± 0.10)	0.24 (± 0.09)
Methylhistidine	1.25 (± 0.03)[b]	1.10 (± 0.19)[a,b]	0.93 (± 0.07)[a]
Ornithine	2.72 (± 0.42)[a,b]	2.27 (± 0.33)[a]	3.26 (± 0.65)[b]
Phenylalanine	1.46 (± 0.20)[b]	1.29 (± 0.19)[a]	1.41 (± 0.03)[a,b]
Phosphocreatine	7.12 (± 2.58)[a]	5.46 (± 1.30)[a]	15.1 (± 7.74)[b]
Proline	15.6 (± 0.85)[a]	14.7 (± 0.61)[a]	18.9 (± 0.41)[b]
Putrescine	0.06 (± 0.01)[b]	0.06 (± 0.02)[b]	0.03 (± 0.02)[a]
Sarcosine	0.50 (± 0.07)	0.48 (± 0.07)	0.41 (± 0.06)
Serine	22.4 (± 2.15)[c]	18.3 (± 0.60)[b]	10.0 (± 0.85)[a]
Serotonin	0.01 (± 0.00)	0.01 (± 0.00)	0.01 (± 0.00)
Spermidine	0.54 (± 0.27)	0.49 (± 0.23)	0.48 (± 0.27)
Spermine	0.21 (± 0.20)	0.21 (± 0.21)	0.26 (± 0.24)
Symmetric	4.04 (± 0.54)[b]	3.08 (± 0.25)[a]	2.98 (± 0.30)[a]

dimethylarginine			
Taurine	27.3 (± 1.72)[a,b]	26.4 (± 1.41)[a]	30.1 (± 0.38)[b]
Threonine	4.06 (± 1.61)[b]	3.03 (± 1.36)[a]	3.69 (± 1.65)[a,b]
Total dimethylarginine	4.15 (± 0.59)[b]	3.13 (± 0.24)[a]	3.09 (± 0.28)[a]
trans-4-Hydroxyproline	4.38 (± 0.33)	4.18 (± 0.21)	4.49 (± 0.06)
Trimethylamine N-oxide	3.00 (± 0.40)	3.01 (± 0.22)	3.19 (± 0.10)
Tryptophan	0.72 (± 0.19)	0.68 (± 0.10)	0.71 (± 0.10)
Tyramine	0.01 (± 0.00)	0.01 (± 0.00)	0.01 (± 0.00)
Tyrosine	0.33 (± 0.08)[a,b]	0.28 (± 0.00)[a]	0.43 (± 0.24)[b]
Valine	7.45 (± 0.76)[a]	7.56 (± 0.34)[a]	11.09 (± 0.66)[b]

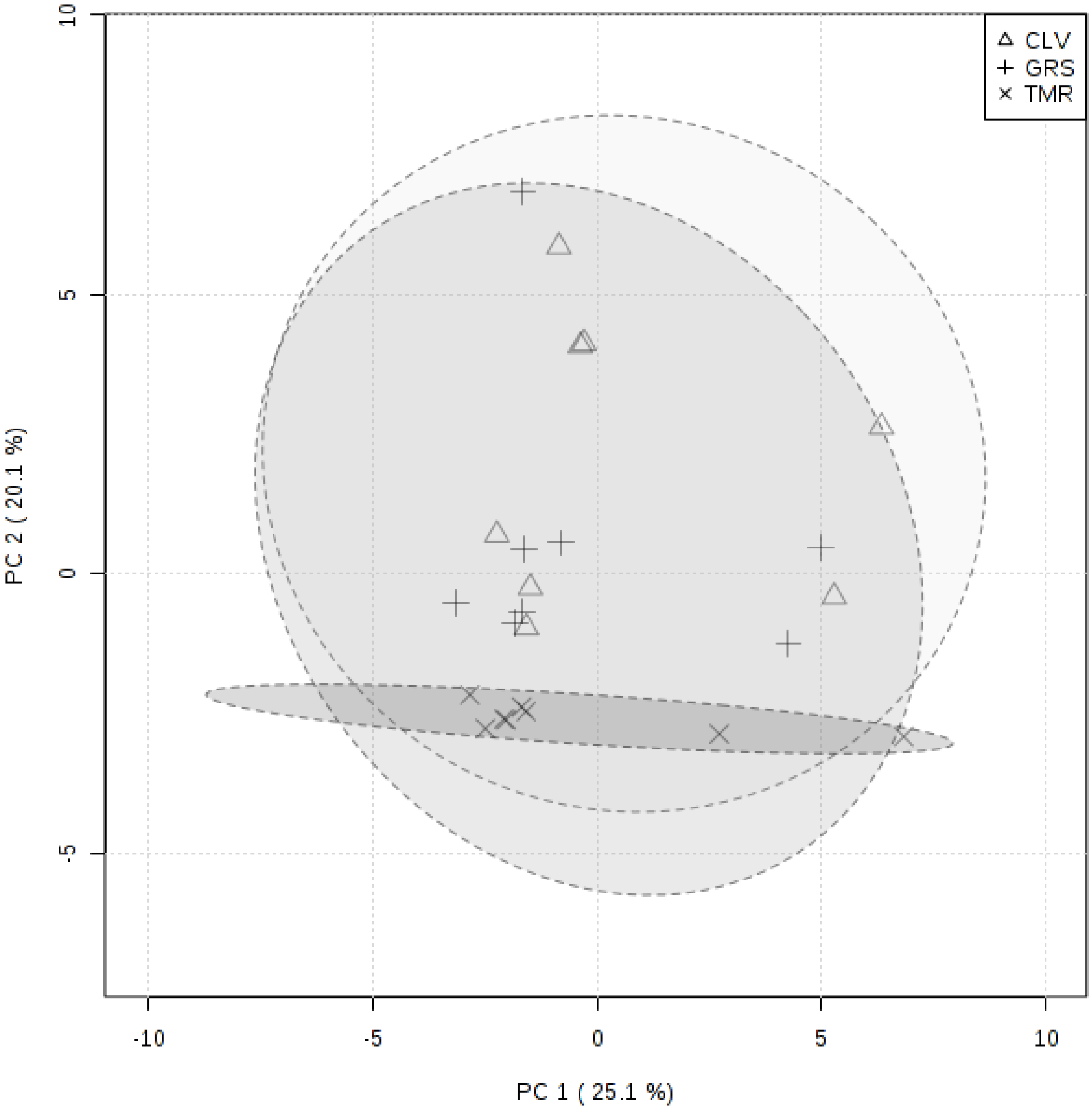

Figure S1. Principal component analysis (PCA) score plot for metabolomics analysis of protein ingredients from perennial ryegrass (GRS), perennial ryegrass/white clover (CLV) and total mixed ration (TMR) feeding systems, determined by LC-MS/MS.
